# Jungfraujoch: 38 GB s^−1^ real-time serial crystallography – data acquisition and live indexing feedback

**DOI:** 10.1107/S1600576726008083

**Published:** 2026-09-30

**Authors:** J. Duan, H.-C. Stadler, J. Nan, Z. Matěj, G. Gotthard, F. Dworkowski, M. Bjelcic, O. Aurelius, A. Mozzanica, S. Grimm, M. Burian, T. Ursby, M. Wang, F. Leonarski

**Affiliations:** a Center for Photon Science Paul Scherrer Institute Forschungsstrasse 111 Villigen 5232 Switzerland; b PSI Center for Scientific Computing, Theory and Data, Paul Scherrer Institute, Forschungsstrasse 111, Villigen, 5232, Switzerland; c MAX IV Laboratory, Lund University Fotongatan 2 Lund 224 84 Sweden; d DECTRIS Ltd, Täfernweg 1, Baden, 5405, Switzerland; Deutsches Elektronen Synchrotron, Germany

**Keywords:** high-throughput data processing, data reduction, macromolecular crystallography, kHz data acquisition, data triage, serial crystallography

## Abstract

Significant growth in the quantity of data presents a critical challenge for macromolecular crystallography at fourth-generation synchrotrons. We introduce an updated version of the Jungfraujoch server, reaching a data throughput of 38 GB s^−1^ from a 9 Mpixel JUNGFRAU detector (JUNGFRAU 9M) operating at 2 kHz, and improving data analysis capabilities with spot finding and indexing. These capabilities provide real-time information that can inform experimental decisions, including which data to retain on disk. In a serial crystallography demonstration at MicroMAX, we illustrate how live indexing integrates into the data collection workflow and show that its results are consistent with offline analysis. By enabling operation of Jungfraujoch on modern x86 hardware, we are lowering the barrier to adopting the system at sources beyond Swiss Light Source 2.0 and MAX IV.

## Abstract 2

Jungfraujoch is a single-node FPGA–GPU server that enables 38 GB s^−1^ real-time crystallographic data acquisition using the PSI JUNGFRAU 9M detector at 2 kHz, with simultaneous on-the-fly processing, spot finding and indexing, and optional adaptive data reduction during continuous data streaming.

## 1. Introduction

For most of synchrotron science’s history, the limiting factor was the instrument: beam brightness, detector sensitivity and readout speed set the ceiling on what could be measured. That ceiling has now been broken. Fourth-generation, diffraction-limited storage rings deliver photon fluxes orders of magnitude beyond what their predecessors could achieve (Denes & Schmitt, 2014 ▸), and a new generation of kilohertz-capable pixel-array detectors can capture structural dynamics that were entirely invisible a decade ago (Broennimann *et al.*, 2006 ▸; Dinapoli *et al.*, 2011 ▸; Mozzanica *et al.*, 2018 ▸; Fröjdh *et al.*, 2024 ▸; Rota *et al.*, 2024 ▸; Nishino *et al.*, 2025 ▸). The bottleneck has moved: our ability to handle the data limits the experimental capability and scientific discovery and application. Synchrotron crystallography is not alone in confronting this shift – genomics, astronomy and scientific imaging face the same reckoning, with data streams routinely reaching tens of gigabytes per second (Price, 2020 ▸; Clissa *et al.*, 2023 ▸) – but in macromolecular crystallography the tension is especially acute. Here real-time, scientifically reliable feedback and data reduction have become as essential to the experiment as the beam itself (Thayer *et al.*, 2024 ▸; Sobolev *et al.*, 2024 ▸; Hu *et al.*, 2024 ▸; Thayer *et al.*, 2017 ▸; White *et al.*, 2025 ▸; Rahmani *et al.*, 2023 ▸; Orlans *et al.*, 2025 ▸).

Jungfraujoch is designed as a single-server appliance combining FPGA and GPU acceleration to control the detector, acquire the incoming raw data stream, convert it into calibrated diffraction images and annotate each image with scientifically relevant information.

Previously, the system ran on IBM POWER9 servers and focused primarily on data acquisition and detector corrections, with throughput limited to approximately 17 GB s^−1^ using a JUNGFRAU 4M detector at 2 kHz (Leonarski, Brückner *et al.*, 2023 ▸; Leonarski, Nan *et al.*, 2023 ▸). A subsequent study demonstrated that GPU-accelerated indexing at kilohertz frame rates is feasible, but real-time usability was constrained by a batching requirement: sustained throughput was only guaranteed when multiple frames were analyzed together rather than individually (Gasparotto *et al.*, 2024 ▸).

Here we present the second generation of the system, addressing each of these limitations in turn. The IBM POWER architecture has been replaced by a mainstream x86 platform, broadening accessibility and simplifying deployment. Spot finding has been reimplemented directly in FPGA firmware for robust, deterministic performance at the full data rate. The GPU indexer has been rewritten in CUDA and the batching constraint eliminated, enabling truly frame-by-frame real-time indexing. Together, these advances double the sustained throughput of the previous system, enabling continuous operation of the JUNGFRAU 9M detector at 2 kHz. To illustrate how these capabilities integrate into a real experiment, we present a serial crystallography demonstration with a high-viscosity extruder (HVE) (Shilova *et al.*, 2020 ▸) conducted at the MicroMAX beamline of MAX IV (Gonzalez *et al.*, 2025 ▸).

## 2. New features in Jungfraujoch

Since the previous reports on Jungfraujoch (Leonarski, Brückner *et al.*, 2023 ▸; Leonarski, Nan *et al.*, 2023 ▸), which focused on capturing diffraction images at kilohertz frame rates with the JUNGFRAU 4M detector and performing detector gain and pedestal corrections, the system has been substantially extended. This section summarizes the major changes.

### 2.1. Transfer to modern x86 server architecture

The initial iteration of Jungfraujoch was developed on IBM servers equipped with the POWER9 processor, which offers unique capabilities for integrating custom accelerators in a memory-coherent way. This architecture enabled rapid FPGA prototyping with limited hardware design experience and was central to the first successful demonstration of the system. However, POWER9 servers also carried the inherent risks of niche hardware: only a single server model (IBM IC922) was compatible, and IBM subsequently decided not to release a direct successor with the POWER10 processor and dropped support to OpenCAPI. A transition to a new architecture was therefore unavoidable. To avoid similar vendor lock-in in the future, we migrated to mainstream x86 servers, where hardware is widely available from multiple vendors and architectural updates are frequent.

To facilitate the transfer, we had to modify the FPGA design and replace the OpenCAPI block used for communication with IBM POWER with a Xilinx direct memory access (XDMA) block for the PCI Express bus. The use of XDMA introduced additional constraints on memory management compared with OpenCAPI. In particular, XDMA operates in the physical address space, whereas OpenCAPI exposes a virtual-address interface. Physical addresses are managed by the operating system kernel and are not directly visible to user-space applications, while virtual addresses are used at the application level and provide a more flexible programming model. To accommodate these requirements, we implemented physically contiguous, cache-coherent buffers in kernel space using the Linux dma_alloc_coherent() API, managed by a custom device driver and mapped into user space via mmap(). Since this transition required substantial changes to the FPGA and host interface, we also took the opportunity to address several other limitations in the existing FPGA design, as described in the following section.

### 2.2. Updated FPGA design

The change in host processor architecture necessitated replacing the FPGA board. We adopted the Xilinx/AMD Alveo U55C, which offers triple the logic resources and twice the high-bandwidth memory (HBM) capacity compared with the previous Alpha Data 9H3 card. The increased HBM capacity enabled the introduction of a packet buffer, where incoming detector data are first staged in HBM before being forwarded to the host CPU. This makes the acquisition pipeline substantially more robust against transient stalls in host-side processing. The data acquisition firmware was also extended to support PSI EIGER packet formats – including 8-bit, 16-bit and 32-bit output modes – in addition to JUNGFRAU.

**Figure 1:**
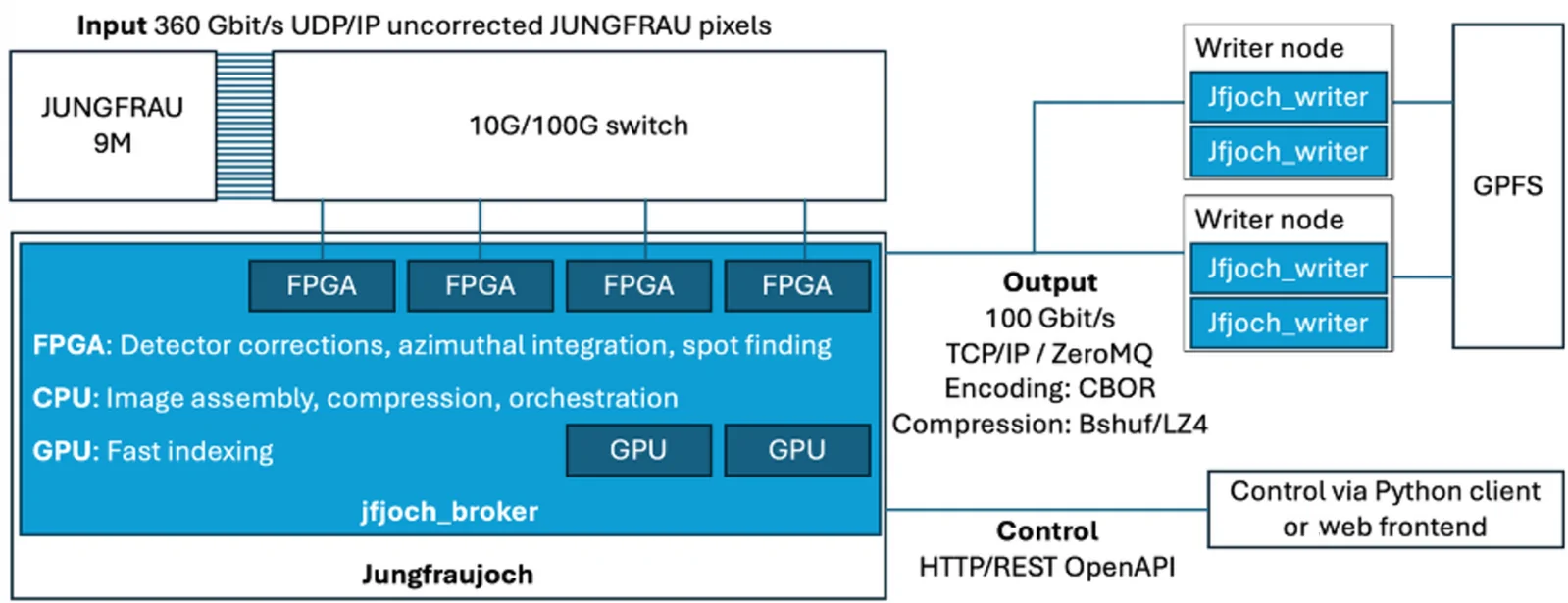
Schematic of Jungfraujoch integration with JUNGFRAU 9M and beamline infrastructure. Middle part presents the Jungfraujoch server, running the *jfjoch_broker* executable to manage FPGA and GPU accelerated tasks. Dedicated writer nodes, connected to facility GPFS (data storage), run *jfjoch_writer* processes to save data in HDF5 files.

The FPGA design supports two network configurations; a detailed schematic is presented in Fig. 1 ▸. The first uses a single 100 Gigabit Ethernet (100GbE) block, which provides maximum bandwidth but requires an intervening network switch to aggregate the detector links. The second uses two 4 × 10GbE blocks, providing eight 10GbE links that can connect directly to the detector without a switch, at an aggregated bandwidth of 8 × 10GbE. The choice of configuration is therefore driven by the available network infrastructure: the 100GbE variant is preferred where a switch is present, while the 8 × 10GbE variant enables simpler direct-connect deployments.

The most significant additions to the FPGA design, described in the following subsections, are on-board data processing capabilities: spot finding and fast azimuthal integration. Both exploit a key property of FPGA-based implementations – once a function fits within the available fabric, it executes at the full detector line rate with negligible latency overhead, independently of CPU or GPU load.

#### 2.2.1. Spot finding

Spot finding in Jungfraujoch follows a two-stage procedure modeled after *COLSPOT* (Kabsch, 2010 ▸). In the first stage, implemented on the FPGA, each pixel is classified as strong if its intensity exceeds a threshold defined as the local mean plus a user-defined multiple of the local standard deviation, both computed within a neighborhood centered on that pixel. In conventional software implementations this neighborhood is typically a 7 × 7 window; our experience with weak serial crystallography data suggests that larger neighborhoods improve robustness at very low photon flux.

Implementing a true sliding-window accumulation on the FPGA is constrained by the clock frequency of 200 MHz, which requires transferring 32 horizontally adjacent pixels per cycle. A fully sliding horizontal window would demand a complex multi-accumulator scheme with high resource utilization and tight timing. We instead adopt a hardware-efficient approximation: each 1024-pixel detector row is partitioned into fixed horizontal segments of 32 pixels, while the vertical window remains fully sliding. The resulting detection region is a fixed 32-pixel horizontal band combined with a vertically sliding window of ±15 lines, giving an effective neighborhood of 32 × 31 pixels. In addition to signal-to-noise threshold, we can also restrict strong pixels to a given raw photon count limit.

In the second stage, strong pixels identified by the FPGA are grouped into Bragg spots on the CPU using a single-pass connected-component labeling algorithm optimized for sparse images (Hennequin *et al.*, 2019 ▸).

#### 2.2.2. Azimuthal integration

Within Jungfraujoch, azimuthal integration serves primarily as a real-time diagnostic. By monitoring the mean background intensity in the solvent diffraction ring in the range *d* = 3–5 Å, the system enables continuous HVE alignment by maximizing this background signal and provides early warning of experimental anomalies such as beam loss or sample depletion.

For this application, speed takes priority over the sub-pixel accuracy required for data reduction, and we developed an FPGA-native histogram-based scheme. Each pixel is assigned to one of 2048 radial bins stored in on-chip block RAM. An optional per-pixel correction constant, applied as a multiplicative factor before accumulation, enables corrections for solid angle and polarization consistent with the implementations in *MatFRAIA* and *Azint* (Jensen *et al.*, 2022 ▸). The detector geometry follows the *PyFAI* point-of-normal-incidence (PONI) convention (Ashiotis *et al.*, 2015 ▸). The FPGA implementation comes with strong constraints on what is possible. Pixel splitting is not supported. Because only the mean intensity is accumulated and not the sum of squared intensities, no error estimate is available.

#### 2.2.3. Region-of-interest statistics

Region-of-interest (ROI) statistics complement azimuthal integration and target experiments such as monitoring the decay of an individual Bragg reflection or scanning transmission X-ray microscopy (STXM). Each detector pixel can be assigned to any of 16 ROIs, and a single pixel may belong to several regions simultaneously. For every ROI the firmware accumulates the sum of pixel intensities, the sum of squared intensities and the intensity-weighted pixel coordinates, from which the mean intensity, standard deviation and center of mass are recovered in real time. Although the user interface defines ROIs as rectangles, circles or azimuthal sectors, the FPGA assigns pixels to regions through a per-pixel membership bitmap and therefore supports regions of arbitrary shape.

### 2.3. Fast feedback indexing

The final step is indexing, which involves finding a crystal lattice that matches the pattern of Bragg spots identified in the previous step. At Jungfraujoch, indexing is implemented using the *Fast Feedback Indexer*, a C++ and CUDA implementation of the TORO algorithm (Gasparotto *et al.*, 2024 ▸) highly optimized for online processing. This approach enables sub-millisecond indexing of diffraction patterns with a known unit cell.

The primary advantage of this implementation over the original *PyTorch* (https://pytorch.org/) TORO version is its ability to achieve kHz performance by processing individual frames, rather than relying on batch processing as required by TORO. In the *Fast Feedback Indexer*, three stages – vector sampling, vector refinement and cell sampling – are executed on the GPU, while a final stage (cell refinement) runs on the CPU, leveraging the *Eigen* template library for linear algebra (Guennebaud *et al.*, 2010 ▸).

To maintain high throughput, computations for separate frames can run independently and in parallel. The *Fast Feedback Indexer* is currently available in *CrystFEL* (White *et al.*, 2012 ▸; White *et al.*, 2016 ▸) and *DIALS* (Winter *et al.*, 2018 ▸).

To complement it for situations where the unit cell is unknown, we have also reimplemented the classical Rossmann indexing algorithm (Powell, 1999 ▸; Steller *et al.*, 1997 ▸). The algorithm runs on the GPU and is implemented using the *cuFFT* library (https://developer.nvidia.com/cufft); subsequent refinement steps are shared with the *Fast Feedback Indexer*.

### 2.4. Compression

By default, Jungfraujoch applies lossless compression using the bitshuffle filter combined with either LZ4 or Zstandard (Masui *et al.*, 2015 ▸) – the de facto community standard for pixel-array detector data, and the scheme used in our previous work. In addition, two optional, application-specific lossy schemes are available.

The first is a quantization inspired by approaches used in ptychography. Each pixel value P is transformed to round(SQ × √P), equivalently round[√(SQ^2^ × P)], where SQ is a small integer scaling factor (typical values 2–4). Because the transform acts on the square root of the intensity, it stabilizes the Poisson variance while reducing the bit width of the data – useful, for example, when streaming data to a GPU for tomographic reconstruction. This scheme is implemented in the FPGA but has low relevance regarding serial crystallography.

The second scheme is designed for serial crystallography cases, where low sample hit rates produce large numbers of images containing no useful diffraction. It resembles existing spot-count-based data reduction approaches (Barty *et al.*, 2014 ▸; Galchenkova *et al.*, 2024 ▸), but goes one step further by using the indexing outcome as the acceptance gate: every image that yields an indexing solution is retained, together with a small, randomly selected fraction of non-indexed images (*e.g.* 5%) that serve as a control set for the quality checks of the gate.

### 2.5. CBOR streaming infrastructure

Jungfraujoch requires a well defined interface between the acquisition server and the downstream consumers – including the HDF5 file writer, beamline control systems and external processing tools – with low latency and sufficient per-image metadata for archiving and real-time analysis. As no widely adopted community standard exists for this purpose, we adopted the DECTRIS *Stream2* interface (Burian *et al.*, 2023 ▸; Donath *et al.*, 2023 ▸) as the foundation of this communication layer. *Stream2* encodes all messages using CBOR (concise binary object representation, RFC 8949) and delivers them through DECTRIS-compatible ZeroMQ PUSH sockets. In addition, we developed a custom Jungfraujoch TCP/IP interface that provides acknowledgements for both image receipt and successful image storage. This mechanism improves robustness by allowing downstream components to report failures, such as insufficient disk space or missing write permissions, back to the acquisition system.

The CBOR-based communication protocol defines three message types compatible with the DECTRIS specification: a start message carrying the complete acquisition configuration, a per-image message containing compressed pixel data and analysis annotations, and an end message summarizing run-level statistics. Jungfraujoch extends this protocol with a fourth message type, the calibration message, which is used to transmit pedestal arrays.

Jungfraujoch implements a superset of the DECTRIS *Stream2* specification. In addition to the standard fields, each image message is enriched with the outputs of the on-the-fly analysis pipeline, including spot positions and intensities, spot counts at each filtering stage, azimuthal integration profiles, indexing results and lattice parameters, and per-image diagnostics such as background estimates, saturated-pixel counts and data collection efficiency metrics.

To maximize data throughput, Jungfraujoch can distribute acquisition data across multiple independent streams. Each stream carries its own start and end messages together with a subset of the image messages. This approach enables multiple independent HDF5 writer processes to operate in parallel, achieving high aggregate write rates without relying on more complex parallel HDF5 implementations.

We provide a dedicated HDF5 writer, *jfjoch_writer*, which consumes the CBOR stream produced by Jungfraujoch and generates NXmx-compliant raw data files (Bernstein *et al.*, 2020 ▸; Könnecke *et al.*, 2015 ▸). To improve robustness in the presence of storage-related failures, we also developed a custom bypass HDF5 virtual file driver (VFD). This driver enables HDF5 file handles to be closed cleanly after input/output errors, facilitating graceful recovery from conditions such as quota exhaustion or insufficient disk space.

### 2.6. HTTP/OpenAPI control and diagnostics interface

The Jungfraujoch broker service (*jfjoch_broker*) orchestrates operation of the FPGA, GPU and outgoing network sockets. It exposes an HTTP-based control interface described by an OpenAPI specification. The specification serves as the authoritative description of all available operations, and is used both to generate the server-side stub code within Jungfraujoch and to produce client libraries. We publish a Python client generated directly from this specification on *PyPI*.

**Figure 2:**
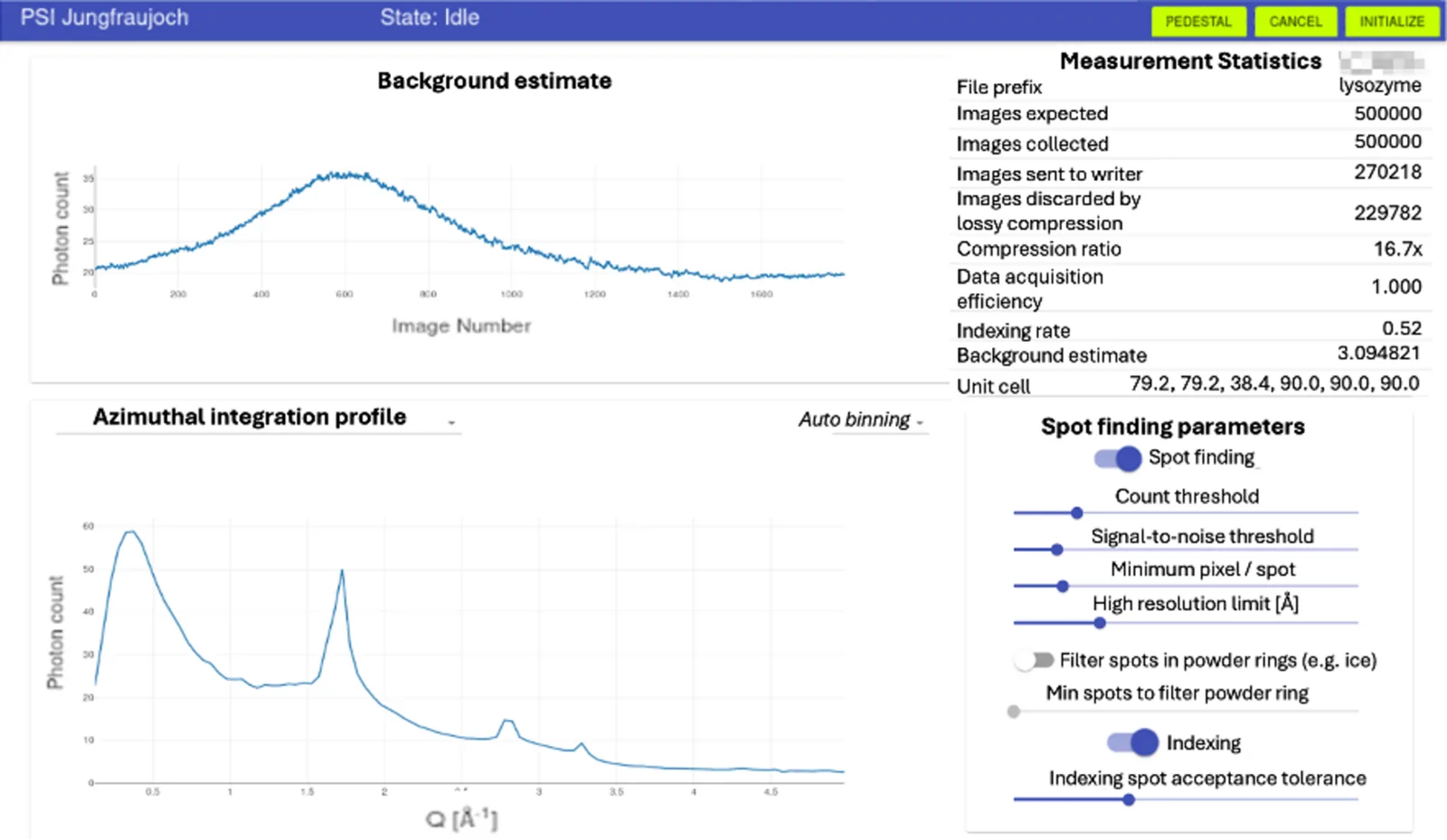
Jungfraujoch: main page of the web frontend. The web frontend visualizes the current state of the detector and data acquisition system (top status bar), shows data acquisition live plots (left part), presents measurement statistics, like images collected and average compression ratio (top right), and allows the user to adjust spot finding parameters during data collection using sliders (bottom right).

To simplify integration in new beamline environments, we have developed a web frontend written in TypeScript/React that communicates directly with the HTTP API. Through this interface (Fig. 2 ▸), users can configure data acquisition and analysis parameters, preview diffraction images, and inspect per-run statistics such as spot counts, azimuthal integration profiles and indexing results. An additional benefit of the OpenAPI approach is that documentation can be generated automatically from the specification, lowering the barrier for new users and instrument integrators.

### 2.7. Upgrade from 4M to 9M pixels

In parallel with the architectural changes described above, the detector itself was upgraded from the JUNGFRAU 4M to the JUNGFRAU 9M, increasing the raw data rate from 17 to 38 GB s^−1^, a factor of 2.25. Sustaining this throughput required increasing the number of FPGA boards per server from two to four, as well as targeted optimization of the CPU-side codebase to eliminate processing bottlenecks identified under the higher load.

## 3. Serial crystallography demonstration at MicroMAX

### 3.1. Macromolecular serial crystallography workflow with real-time data processing

**Figure 3:**
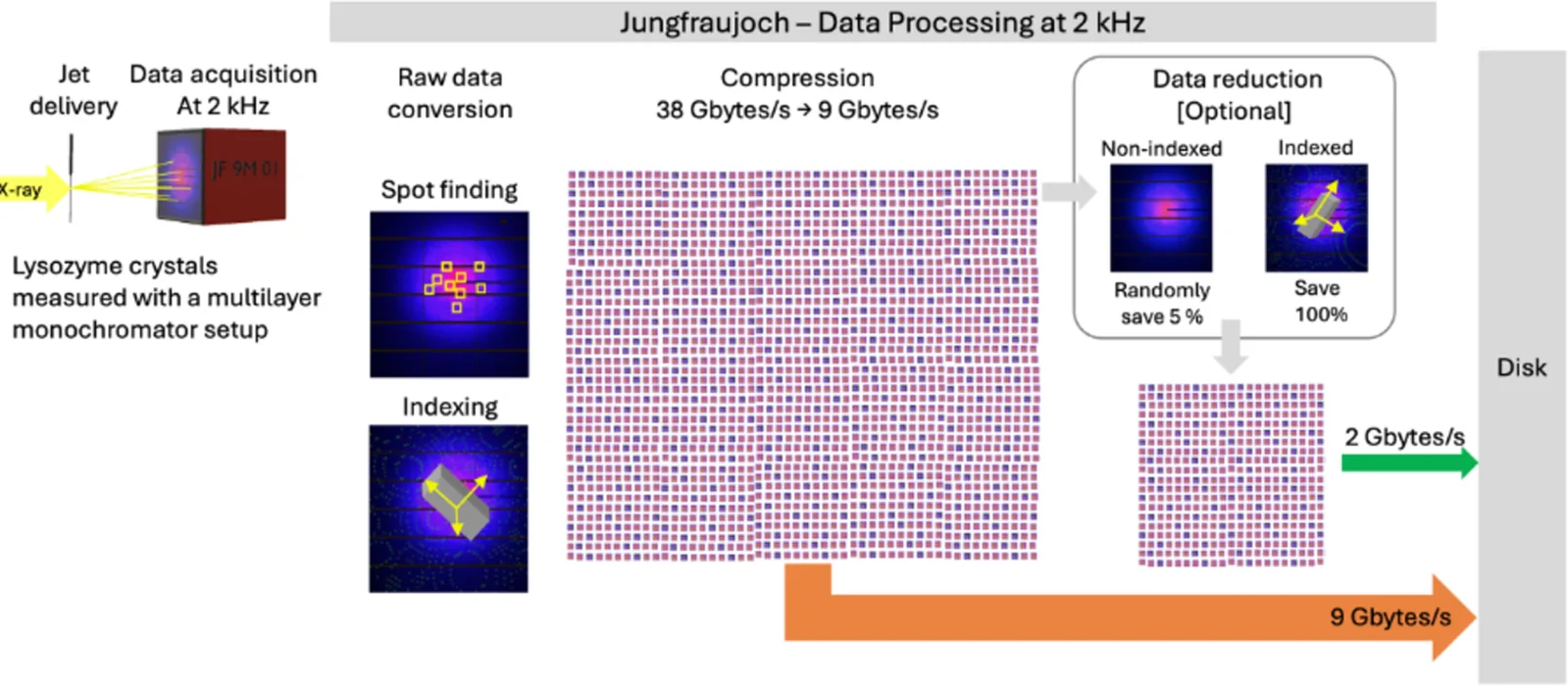
Illustration of data acquisition and data processing in serial macromolecular crystallography. Each of the thumbnails in the mosaic region represents a single-shot diffraction image.

In this section, we illustrate how the real-time analysis capabilities described above integrate into a serial crystallography workflow at a fourth-generation synchrotron. The workflow from data acquisition to data storage is shown in Fig. 3 ▸. Lysozyme crystals were mixed with a medium and then loaded into an HVE for delivering the sample to the X-ray path (Appendix *A*[App appa]). Using either the beamline control interface or the Jungfraujoch web interface, a specified number of triggers was sent to the detector, initiating data acquisition. In our experiments, individual acquisitions ranged from 100000 to 500000 images. Real-time visual monitoring at the beamline, combined with immediate feedback from the Jungfraujoch web interface, enables dynamic control of the experiment. Data collection can be halted when issues such as HVE clogging or sample depletion are observed, or when sample changing is necessary due to insufficient quality.

The system sustained 2 kHz performance from data acquisition through data processing. Because indexing results are available in real time, the system can optionally apply a configurable data reduction filter to select which frames are written to disk (see Section 2.4). In this demonstration, we configured the filter to retain all indexed frames together with a random subset of non-indexed frames (5% in this work). The latter served as an unbiased reference for later verification. The specific retention fraction and filter criteria can be adjusted to match the needs of a given experiment. For beamlines where storage bandwidth or capacity is a constraint, such filtering can substantially reduce the volume of data written to disk. In a case with a highly concentrated lysozyme sample yielding an indexing rate of about 17%, the filter reduced the written data volume by roughly a factor of four. The degree of reduction will vary with sample, concentration and filter configuration, and not all facilities will face storage limitations and writing bandwidth challenges; therefore, the filter is provided as an option, not a requirement.

### 3.2. Consistency between the real-time and offline indexing

To assess how closely the Jungfraujoch live indexing agrees with established offline analysis, we compared indexing outcomes on two test datasets in which all frames were saved without filtering. One dataset was collected with a double-crystal monochromator (DCM), yielding an indexing rate of ∼50%, and the others with a double multilayer monochromator (DMM), with an indexing rate around 15.8% for one HVE in run2 and around 36.8% for another HVE in run10 operated at different speeds. All datasets were independently processed offline with *CrystFEL 0.10.2* (White *et al.*, 2012 ▸) using the Peakfinder8 algorithm (Barty *et al.*, 2014 ▸) for spot finding and *XGandalf* (Gevorkov *et al.*, 2019 ▸) for indexing (Appendix *B*[App appb]) with optimized parameters (Appendix *C*[App appc]). We acknowledge that the *CrystFEL* parameters reflect individual choices, and thus a different expert may optimize the parameters further. Thus, neither method should be regarded as definitive ground truth. Nonetheless, they serve as reasonable grounds for evaluating the consistency between real-time and offline analysis.

For the DCM dataset, the two methods show strong agreement: the fractions on which they disagree are below 2% in both directions (Table 1 ▸), likely reflecting the inherent ambiguity in optimizing spot finding and indexing parameters. The recall is calculated by dividing the true positive by the total sum of positive instances. In Table 1 ▸, it is 49736/(49736 + 2027) ≃ 96.1%.

For the DMM dataset (Table 2 ▸), the agreement on indexed frames is particularly close – around 0.2% of frames flagged as non-indexed by Jungfraujoch can be indexed by *CrystFEL*, indicating that very few diffraction patterns of interest would be missed by the live filter. The large number of frames indexed by Jungfraujoch but not by *CrystFEL* reflects deliberately conservative filter criteria: the system was intentionally biased towards false positives, favoring data retention over the risk of discarding potentially useful frames.

## 4. Data formats and FAIR compliance

The goal of the Jungfraujoch framework is not only to store high-throughput datasets efficiently but also to ensure that these data are findable, accessible, interoperable and reusable (FAIR) (Wilkinson *et al.*, 2016 ▸). Findability of serial crystallography data is often hindered by a lack of descriptive metadata characterizing dataset contents. Parameters such as background level, number of diffraction spots or indexing outcomes can enable users to assess dataset quality and relevance prior to inspecting the raw images.

Although raw diffraction datasets may be formally accessible through persistent identifiers and standardized retrieval mechanisms, practical access to experiments comprising tens to hundreds of terabytes remains challenging. Standard retrieval protocols such as HTTP provide dataset availability but do not offer efficient mechanisms for selective inspection or evaluation of data at this scale. As a result, users often must download substantial fractions of a dataset before determining its relevance or quality. Derived metadata and reduced representations therefore play an important role in enabling efficient discovery, assessment and reuse of these datasets.

Jungfraujoch addresses these challenges by integrating data acquisition with real-time analysis. Because these capabilities are used to guide experiments through rapid feedback and data reduction, transparency and reproducibility of image processing are essential. At a minimum, spot finding and indexing results should be preserved together with detailed information on the applied filters. In addition, unbiased, downscaled reference datasets containing unfiltered images should be retained to enable validation of processing decisions and facilitate data reuse.

For storage of spot finding and indexing results, we adopted a data layout similar to that used by the Coherent X-ray Imaging (CXI) Data Bank (Maia, 2012 ▸). In this approach, spot properties such as position, intensity and indexing information are stored in fixed-size arrays indexed by image number. Each image is allocated space for up to a predefined maximum number of spots, resulting in dense two-dimensional datasets that can be accessed efficiently using standard HDF5 indexing operations. Although this approach introduces some storage overhead due to unused array elements, it enables rapid retrieval of spot information for individual images without requiring traversal of variable-length structures.

We also evaluated the use of the NeXus NXreflections base class. The NXreflections model is well suited to integrated reflection data, where reflections are naturally represented as a dataset-wide table. This approach aligns closely with conventional rotation crystallography workflows and is appropriate for storing three-dimensional integration results. However, spot finding and indexing results in serial crystallography are inherently image centric. In datasets containing hundreds of thousands of diffraction patterns, representing all spots in a single reflection table requires aggregation of information across the entire experiment before efficient access to the spots associated with an individual image is possible.

For this reason, we consider a per-image representation, similar to that employed by CXI and supported by *CrystFEL*, to be more suitable for intermediate serial crystallography results such as spot finding and indexing. We encourage the community to develop standardized NeXus application definitions for these data products, combining the interoperability benefits of NeXus with data structures that reflect the access patterns and scale of modern serial crystallography experiments.

## 5. Future outlook

Stable data acquisition and indexing already provide a strong foundation for a fully integrated live-processing system. Our long-term goal is to further extend Jungfraujoch into an end-to-end pipeline that converts X-ray diffraction images directly into merged reflection intensities. The main processing stages currently under development are geometry refinement, integration, scaling, merging and symmetry determination.

Data rates at synchrotron and Xray free electron laser facilities continue to grow rapidly (Leonarski, Brückner *et al.*, 2023 ▸; Galchenkova *et al.*, 2024 ▸; Dimper *et al.*, 2019 ▸; Thayer *et al.*, 2024 ▸; Li *et al.*, 2023 ▸) and no single reduction strategy fits every experiment – gating empty frames is one route, retaining only regions around reflections or sparsified frames (Underwood *et al.*, 2023 ▸; Kieffer *et al.*, 2025 ▸) is another, and keeping merged intensities while discarding images a third. Since each of these depends on analyzing frames on the fly, and since effective reduction in macromolecular crystallography calls for schemes tailored to diffraction data rather than generic compressors (Galchenkova *et al.*, 2024 ▸), we regard Jungfraujoch’s contribution as providing the real-time, line-rate analysis on which such application-specific reductions can be built.

The future direction is also moving from a single application performing detector data acquisition to an ecosystem of software maximizing output of the X-ray detectors at macromolecular crystallography beamlines. We are extending Jungfraujoch to enable analysis of data streamed by DECTRIS detector control unit servers. We are also developing a diffraction viewer to visualize all information annotated by Jungfraujoch and offline analysis tools, allowing users to re-apply Jungfraujoch processing methods after the experiment.

Two generations of Jungfraujoch development have clarified the role FPGAs should play in detector data acquisition. For frontier detectors, where the aim is to push frame rates to the limit of what is physically achievable, FPGAs remain indispensable: only purpose-built hardware delivers the deterministic, real-time throughput these detectors demand, independently of host load. At the same time, custom hardware is a persistent obstacle to deployment at scale and to long-term availability – FPGA designs are bound to specific boards and toolchains, demand specialized expertise, and age poorly as vendors discontinue products, as our forced migration away from IBM POWER illustrates. A commodity x86/NVIDIA GPU server, by contrast, is currently the simplest system to deploy and sustain and should be the baseline for any broadly applicable installation; the price is performance, since a CPU/GPU pipeline can approach but never fully match the hard real-time behavior of an FPGA. We therefore see no single winner and instead pursue both routes in parallel – an FPGA-accelerated configuration for the most demanding, rate-limited experiments and a portable CPU/GPU configuration for broad deployment – keeping the data acquisition and analysis software able to target either workflow.

## 6. Jungfraujoch availability

We encourage interested readers to visit the Gitea repository (https://gitea.psi.ch/mx/jungfraujoch) to acquire the newest version of the software. Specifically, at the time of writing, we provide pre-built packages for RHEL8, RHEL9, Ubuntu 22.04 and Ubuntu 24.04, as well as synthesized FPGA images for the AMD U55C card. Jungfraujoch software is licensed under the GPLv3 license. Jungfraujoch FPGA is licensed with the CERN OHL-S license.

The CUDA implementation of the TORO indexer, referred to as the *Fast Feedback Indexer*, can be found at https://github.com/paulscherrerinstitute/fast-feedback-indexer. This indexer is also available in *DIALS* and *CrystFEL*.

## Supplementary Material

Raw data (DCM and DMM datasets) used for producing statistics discussed in this paper: https://doi.org/10.48391/32b38db5-dedd-4e8a-aedc-31bd62e93f65

## Figures and Tables

**Table 1:** Consistency of the indexing outcomes between Jungfraujoch live processing and *CrystFEL* offline analysis for the DCM dataset DCM (100000 frames) – run1 (Jungfraujoch index rate 51.2%). Recall 96.1%. Percentages are fractions of the total number of frames.

	Jungfraujoch indexed	Jungfraujoch non-indexed
*CrystFEL* indexed	49736 (49.7%)	2027 (2.0%)
*CrystFEL* non-indexed	1497 (1.5%)	46740 (46.7%)

**Table:** DMM (200000 frames) – run2 (Jungfraujoch index rate 15.8%). Recall 95.1%.

	Jungfraujoch indexed	Jungfraujoch non-indexed 5% filtered	Jungfraujoch non-indexed extrapolated
*CrystFEL* indexed	8537 (4.3%)	21	437 (0.2%)
*CrystFEL* non-indexed	22940 (11.5%)	8472	168106 (84.0%)

**Table:** DMM (500000 frames) – run10 (Jungfraujoch index rate 36.8%). Recall 98.0%.

	Jungfraujoch indexed	Jungfraujoch non-indexed 5% filtered	Jungfraujoch non-indexed extrapolated
*CrystFEL* indexed	44364 (8.9%)	46	906 (0.2%)
*CrystFEL* non-indexed	139488 (27.9%)	15998	315241 (63.0%)

The 5% filtered non-indexed frames were extrapolated (see Appendix *B*[App appb]). Percentages are fractions of the total number of frames.

## Data Availability

Data (DCM and DMM datasets) used for producing statistics shown in this paper can be accessed at https://doi.org/10.48391/32b38db5-dedd-4e8a-aedc-31bd62e93f65. The data processing script can be downloaded from https://github.com/jdawnduan/Jungfraujoch-DCM-DMM-datasets-processing-scripts/archive/refs/tags/v1.0.0.zip.

## Appendix A Sample preparation, delivery and X-ray data collection

The HVE was used to deliver lysozyme samples to the focus of the X-ray beam at MicroMAX. Sample centering and data acquisition were carried out with the beamline control software *MXCuBE3* (Mueller *et al.*, 2017 ▸). Using the OpenAPI-based REST interface, the JUNGFRAU detector was integrated into the beamline control system.

The diffraction data were collected with the PSI-developed adaptive-gain, charge-integrating JUNGFRAU 9M detector (Mozzanica *et al.*, 2018 ▸; Leonarski *et al.*, 2018 ▸). This detector is composed of 18 modules, each of 512 × 1024 pixels, roughly nine million pixels (Mpixel) in total, with a single pixel size of 75 × 75 µm. Data from each module are sent over dedicated fiber-optic links, 20 Gbit s^−1^ per module, totaling 360 Gbit s^−1^ network bandwidth for the whole system. Since a single pixel is encoded with 16 bits and because of the convenience of using a round number, the detector was operated at a 2 kHz frame rate (500 µs frame time, 480 µs count time) and the effective data rate was 38 GB s^−1^ (302 Gbit s^−1^). Detector gain calibration and pedestal factor collection were performed using a procedure outlined previously (Redford *et al.*, 2018 ▸; Leonarski *et al.*, 2020 ▸). To reduce the dark current, the chiller was set to −10 °C, cooling the detector to approximately 1–2 °C.

To prepare for crystallization, lysozyme (Sigma–Aldrich) was dissolved in 100 m*M* sodium acetate, pH 3.0, to a final concentration of 25 mg ml^−1^. To obtain microcrystals, the lysozyme solution was mixed 1:1 with precipitant solution (22% NaCl, 6.4% polyethyl­ene glycol 6000 in 80 m*M* sodium acetate, pH 3.0) and incubated overnight. The resulting crystals had an average size distribution of 20 × 15 × 15 µm and were harvested by centrifugation. Cellulose matrix was prepared by dissolving 22% *w*/*v* 2-hy­droxy­ethyl-cellulose in H_2_O and leaving it to swell overnight. For data collection, the crystals were embedded 1:4 in the cellulose matrix.

The HVE injector (Max Planck Institute for Medical Research, Heidelberg, Germany) (Shilova *et al.*, 2020 ▸) used in this experiment was mounted vertically to the MicroMAX micro-diffractometer (MD3, ARINAX, France). Protein crystals loaded into the HVE reservoir were extruded through tipped silica capillaries with an inner diameter of 75 µm by a high-performance liquid chromatography (HPLC) pump (Shimadzu, Japan, LC-20AD), and the sample HVE was stabilized with helium as sheath gas (Shilova *et al.*, 2020 ▸). The X-ray beam was focused to a 20 × 5 µm FWHM spot at the sample position, and the beam energy was set to 12.99 keV. Two monochromators were used: a double-crystal monochromator and a double multilayer monochromator with bandwidth and flux of ∼0.014%, 5 × 10^12^ ph s^−1^ and ∼1%, 10^14^ ph s^−1^, respectively. Lysozyme microcrystals were extruded at speeds of 2.8 mm s^−1^ for data collection at 2 kHz. For run10 of the DMM dataset, the speed was increased to 5 mm s^−1^.

## Appendix B Data selection and processing details

The frames to be processed were selected using the imageIndexed flag stored in the data h5 file (*e.g.*LysozymeJet5-lysozyme1_2_data_000001.h5/entry/MX/imageIndexed), which records Jungfrau­joch’s real-time indexing decision for each frame. Frames with the flag set to 1 (indexed) were written to the lstind file, and frames with the flag set to 0 (non-indexed) were written to the lst file. A third list file, fulllst, contains the complete dataset. The geometry file was manually optimized.

The two datasets differ in how non-indexed frames were saved to disk. For the DCM dataset, the non-indexed filter was set to 100%, so every frame was saved, including non-indexed ones. For the DMM dataset, the filter was set to 5%, meaning each frame that Jungfraujoch flagged as non-indexed during real-time processing had only a 5% chance of being written to disk.

After separating each dataset into Jungfraujoch-indexed frames (lstind), Jungfraujoch non-indexed frames (lst) and the full dataset (fulllst), *CrystFEL* was run on each list file to count the frames it could actually index. For the DCM dataset, the resulting counts for the lstind and non-indexed files are logged directly in Table 1 ▸. For the DMM dataset, because the saved non-indexed frames represent only ∼5% of the true non-indexed population, the raw *CrystFEL* counts are reported in the middle column of Table 2 ▸, and the corresponding values extrapolated to the full non-indexed population are reported in the last column. The percentages were calculated from the extrapolated counts, so that the common denominator is the number of frames targeted at the time of data collection rather than the number of frames saved to disk.

## Appendix C Optimized *CrystFEL* data processing parameters

*Indexamajig* parameters for processing the DCM dataset:

indexamajig --peaks=peakfinder8 --indexing=xgandalf --xgandalf-fast-execution --threshold=7--min-snr=6 --int-radius=2,3,5 -p lyso.cell --min-peaks=6 --min-pix-count=1 -i fulllst -o fulllst.stream -g dcm.geom -j 32 --min-res=75 --multi --harvest-file=fulllst.json

*Indexamajig* parameters for processing the DMM dataset:

indexamajig --peaks=peakfinder8 --indexing=xgandalf-latt-cell --xgandalf-fast-execution--tolerance=10.0,10.0,10.0,2,3,2 --threshold=45 --min-snr=4 --int-radius=2,3,6 --integration= rings-grad -p lyso.cell --min-pix-count=1 --no-multi -i fulllst -o fulllst.stream -g mlm.geom -j 36 --min-res=85 --check-peaks --max-res=3000 --local-bg-radius=5 --harvest-file=fulllst.json
